# IFN-γ decreases PD-1 in T lymphocytes from convalescent COVID-19 patients via the AKT/GSK3β signaling pathway

**DOI:** 10.1038/s41598-024-55191-6

**Published:** 2024-02-29

**Authors:** Meijuan Song, Xiangqun Liu, Weiyu Shen, Zhengxia Wang, Jingjing Wu, Jingxian Jiang, Yanan Liu, Tingting Xu, Tao Bian, Mingshun Zhang, Wei Sun, Mao Huang, Ningfei Ji

**Affiliations:** 1 Department of Respiratory and Critical Care Medicine, Jiangsu Province People’s Hospital and Nanjing Medical University First Affiliated Hospital, Nanjing, China; 2 Department of Respiratory and Critical Care Medicine, The Xuzhou Municipal Hospital Affiliated to Xuzhou Medical University, Xuzhou, China; 3 Department of Respiratory and Critical Care Medicine, Wuxi People’s Hospital Affiliated to Nanjing Medical University, Wuxi, China; 4 Jiangsu Province Engineering Research Center of Antibody Drug, NHC Key Laboratory of Antibody Technique, Department of Immunology, Nanjing Medical University, Nanjing, China; 5 Department of Respiratory and Critical Care Medicine, Xishan People’s Hospital of Wuxi City, Wuxi, China

**Keywords:** Coronavirus disease 2019, Convalescent patients, CD8^+^ T cells, PD-1, IFN-γ, Checkpoint signalling, Viral infection

## Abstract

Post-COVID-19 syndrome may be associated with the abnormal immune status. Compared with the unexposed age-matched elder group, PD-1 in the CD8^+^ T cells from recovered COVID-19 patients was significantly lower. IFN-γ in the plasma of COVID-19 convalescent patients was increased, which inhibited PD-1 expression in CD8^+^ T cells from COVID-19 convalescent patients. scRNA-seq bioinformatics analysis revealed that AKT/GSK3β may regulate the INF-γ/PD-1 axis in CD8^+^ T cells from COVID-19 convalescent patients. In parallel, an IFN-γ neutralizing antibody reduced AKT and increased GSK3β in PBMCs. An AKT agonist (SC79) significantly decreased p-GSK3β. Moreover, AKT decreased PD-1 on CD8^+^ T cells, and GSK3β increased PD-1 on CD8^+^ T cells according to flow cytometry analysis. Collectively, we demonstrated that recovered COVID-19 patients may develop long COVID. Increased IFN-γ in the plasma of recovered Wuhan COVID-19 patients contributed to PD-1 downregulation on CD8^+^ T cells by regulating the AKT/GSK3β signaling pathway.

## Introduction

Severe acute respiratory syndrome coronavirus 2 (SARS-CoV-2) caused a global outbreak of coronavirus disease 2019 (COVID-19). While many COVID-19 patients recover within weeks, a significant proportion of recovered individuals with COVID-19 experience prolonged physical and cognitive impairments^1^. These phenomena are collectively referred to as the “long COVID-19” phenomenon or the acute sequelae of SARS-CoV-2 infection^2^. The immune status of these patients is largely unmasked.

The severity of SARS-CoV-2 infection is associated with an uncontrolled innate inflammatory response and an impaired adaptive immune response, mainly due to T lymphocyte exhaustion and lymphopenia. The clearance of SARS-CoV-2 is dependent on the effective T-cell response to viral antigens^3^, especially antigen-specific CD8^+^ T cells, which play a key role in eliminating virus-infected cells^4–6^. CD8^+^ T-cell consumption in viral infection is common, and lymphocyte count is negatively correlated with severity^7–10^. Taeschler et al. found that CD4^+^ and CD8^+^ T cells were continuously activated in patients with mild and severe COVID-19 within 12 months after infection. In a group of patients with severe COVID-19, the CD8^+^ T cell count continued to be low during follow-up, and showed obvious phenotype during acute infection, including dysfunctional T cell reaction and signs of excessive production of proinflammatory cytokines^11^. We hypothesize that long COVID-19 may be associated with the functional status of T lymphocytes, which are the most important cells against SARS-CoV-2.

Programmed death receptor 1 (PD-1) is a member of the CD28 family. It is an immunosuppressive molecule expressed in activated T cells and an important target of immunotherapy. It is able to bind to the ligands PD-L1 and PD-L2 and inhibit T-cell activation^12^. Increased PD-1 expression on T cells contributes to immune dysfunction and impaired clearance of viral infection, hospital infection and cancer^13–17^. PD-1-mediated signaling is similarly involved in several types of infections, such as human immunodeficiency virus (HIV) and hepatitis C virus (HCV)^17–19^. In previous studies, it has been pointed out that during chronic infection and cancer, there is a state of inhibition of T-cell activation and promotion of T-cell dysfunction, which is termed T-cell exhaustion^20^. It is mainly manifested in the expression of exhaustion indicators such as PD-1, Tim-3 and Lag3 on T cells. It has been reported that the increase in PD-1 expression levels in T cells of severe and extremely severe COVID-19 patients is a sign of the transition of T cells from an overactivated state to an exhausted state^21,22^. However, a recent study showed that SARS-CoV-2-specific CD8^+^ T cells expressing PD-1 are actually functional^23^. Some studies have confirmed that PD-1, as a key inhibitory molecule, can also inhibit the secretion of IFN-γ from T cells to maintain peripheral tolerance^24^.

Cytokine storms occur in patients during acute COVID-19 infection, including large accumulations of IFN-γ, IL-1β, IP-10, and MCP-1, which may lead to activation of the Th1-cell response^25^. Cytokine storms are related to the severity of COVID-19^26^. Studies have shown that SARS-CoV-2 may have a negative impact on human pancreatic islet function and survival rate through the generation of inflammatory conditions, which may have a direct effect, and may lead to metabolic abnormalities in patients with COVID-19^27^.

IFN-γ is a critical immunomodulator that regulates the innate and adaptive immune responses to pathogen infection^28^. Ding et al. demonstrated that IFN-γ downregulated PD-1 expression and assisted nivolumab in PD-1 blockade effects on CD8^+^ T-lymphocytes in pancreatic cancer^29^. PD-1 deficiency upregulates IFN-γ secretion to promote T follicular helper cell expansion in infection treatment vaccine-immunized mice^30^. However, it is unknown how IFN-γ participates in the regulation of the T-cell response in COVID-19 convalescent and whether it is involved in PD-1 regulating T-cell immune responses.

In this study, the COVID-19 convalescent population was recruited for 6 months to explore the T-cell-mediated immune response, especially the expression of PD-1 and the secretion of IFN-γ. Compared with age-matched unexposed elderly individuals, COVID-19 convalescent patients manifested with decreased PD-1 on CD8^+^ T lymphocytes. In contrast, IFN-γ in the plasma from COVID-19 convalescent patients was increased. Upon stimulation with the spike protein, T cells from COVID-19 convalescent patients produced more IFN-γ. Combined with single-cell RNA-seq (scRNA-seq) bioinformatics and flow cytometry, we further demonstrated that the AKT/GSK3β signaling pathway may be involved in the regulation of IFN-γ on the expression of PD-1 in CD8^+^ T lymphocytes from COVID-19 convalescent patients. This result provides an important basis for mechanistic research on the sequelae of COVID-19 convalescence and provides important information for our follow-up clinical diagnosis and treatment of COVID-19.

## Materials and methods

### Patients

The Human Ethics Committee of the First Affiliated Hospital of Nanjing Medical University approved this study and obtained voluntary written consent from each enrolled patient (2020-SR-549). Fasting venous blood samples from unexposed sex-matched younger healthy controls (18-23Y), age- and sex-matched unexposed elder (55-65Y) healthy controls and COVID-19 convalescent patients infected with Wuhan strain for 6 months (light/common) were collected directly in EDTA (ethylene diamine tetra acetic acid) vacuum blood collection vessels. The basic information comparison of the three groups of subjects is shown in Table 1, including age, gender, BMI, smoking status, and presence of underlying diseases. In addition, the following inclusion criteria were used in the recruitment of the unexposed population: (1) Healthy individuals who had not received any vaccination. (2) Those who had no previous tumor, diabetes, autoimmune system diseases or other immune system-related diseases. (3) Those who had not taken anti-antibiotics recently. (4) Participants who can fully understand the protocol and research information provided by the investigator. (5) Participants who are willing to give informed consent. Participants were excluded based on the following exclusion criteria: (1) Those who were receiving a drug program targeting the immune system. (2) Those who were taking anti-inflammatory, antibacterial or antiviral drugs during study entry. (3) Those who had received medication within 6 months prior to enrollment and major surgery. (4) Pregnant or breastfeeding women. A participant information sheet was provided to all participants and interpreted by the investigator.

**Table 1:** General information of unexposed younger, unexposed elder and convalescent COVID-19 patients.

	Unexposed younger (n = 20)	Unexposed elder (n = 20)	Convalescent COVID-19 (n = 37)
Age (y)	19.89 ± 1.29	60.89 ± 2.68	50.84 ± 12.94
Gender			
Male	10 (50%)	10 (50%)	22 (59.5%)
Female	10 (50%)	10 (50%)	15 (40.5%)
Smoking			
Never	20 (100%)	13 (65%)	28 (75.7%)
Current	0	3 (15%)	8 (21.6%)
Former	0	4 (20%)	1 (2.7%)
BMI (kg/m^2^)	NA	NA	23.84 ± 2.88
Comorbidities			
Hypertension	0	6 (30%)	10 (27%)
Diabetes	0	0	3 (8.1%)
Hyperlipidemia	0	0	2 (5.4%)
Cardiovascular disease	0	0	2 (5.4%)
Cerebrovascular diseases	0	0	1 (2.7%)
Malignancy	0	0	1 (2.7%)
Heart rate	NA	NA	81 ± 12
Pulse rate	NA	NA	98 (96–100)

*NA* not available.

### PBMC extraction and culture

Peripheral blood mononuclear cells (PBMCs) were extracted by Ficoll-Hypaque (Sigma‒Aldrich, St. Louis, MO, USA) and seeded in a precoated 96-well plate (Costar, Australia) at 1 × 10^5^ cells/well. The subjects were divided into the negative control group, anti-CD3 (clone: OKT3, eBioscience)/CD28 (clone: CD28.2, eBioscience) group (the ratio of anti-CD3 and anti-CD28 was 1:1000), S (Spike) protein group (3 μg/ml, Wuhan SARS-CoV-2 strain, #Z03483, GenScript) combined with anti-CD28, and N (Nucleocapsid) protein (3 μg/ml, Wuhan SARS-CoV-2 strain, #Z03480, GenScript) combined with anti-CD28. RPMI-1640 plus 10% heat inactivated fetal bovine serum (FBS, Gibco, Grand Island, NY). The medium was supplemented with 100 units/ml penicillin, 100 μg/ml streptomycin, and 2 mM l-glutamine. Cells were maintained at 37 °C with 5% CO_2_ in a humidified incubator for 48 h.

### Flow cytometry

PBMCs (2 × 10^5^) resuspended in 1 ml phosphate-buffered saline (PBS) at room temperature (RT) were centrifuged (500*g*, 4 °C, 5 min). Add 1 μl Zombie NIR dead-living dye (Biolegend, Cat# 423105), incubate at r.t. for 30 min in the dark. Then, PBMCs were washed with 0.2% BSA (1 ml/tube) and centrifuged (500*g*, 4 °C, 5 min) twice. The cells were incubated in blocking solution (10% human serum + 1% BSA mixture) for 30 min in the dark at 1 ml/tube on ice and centrifuged (500*g*, 4 °C, 5 min) to block nonspecific antibodies. Next, we added 20 μl antibody mixture (100 μl system staining reaction) and incubated the samples at 4 °C for 45 min. Then, the cells were fixed in 0.8% paraformaldehyde (PFA) at 22 °C for 10 min in the dark, washed and resuspended for analysis. Cells were analyzed on a Cytek Aurora flow cytometer using Cytek Spectroflo software. Analysis was performed using FlowJo V10 (Treestar) software.

### Bioinformatics analysis

To characterize the immune characteristics of COVID-19, Ren et al. established COVID-19 single-cell alliance 122 (SC4) in China. SC4 classified 171 COVID-19-seq patients according to the WHO, including 22 mild/moderate symptoms, 54 hospitalized patients with severe symptoms, 95 recovered patients (57 mild/moderate and 38 severe symptoms), and 25 healthy controls. We included 10 cases in the unexposed younger group, 3 cases in the unexposed elder group and 4 cases in the COVID-19 convalescent group for cluster analysis. These samples are consistent with the grouping and age requirements of our experiment. The software used is Seurat V4.0^31^. The GSE158055 data set was downloaded. The low-quality cells and outlier cells were removed. After normalizing the data, scale and principal component analyses were performed. The principal components for dimensionality reduction are selected by the jackstraw algorithm, and nonlinear dimensionality reduction is carried out by the Umap algorithm. Finally, the result of the cluster is obtained. The Wilcoxon test was used for differential expression analysis. Pathway enrichment analysis was performed using Cluster profiler software. According to the immunologic signature enrichment analysis in the Gene Ontology (GO; http://geneontology.org/) and Kyoto Encyclopedia of Genes and Genomes database (KEGG; http://www.genome.jp/kegg/). P values < 0.01 and < 0.05 were considered to be significant in GO and KEGG analyses, respectively.

### ELISPOT quantitative detection of IFN-γ secreted by T cells

PBMCs were incubated with or without anti-CD3/CD28 (1 µg/ml) stimulation for 48 h at 37 °C. IFN-γ-producing cells in PBMCs were detected using an ELISPOT kit (Mabtech). Briefly, single-cell suspensions were seeded onto the antibody-coated plate at a concentration of 1 × 10^5^ cells/well. The plate was then incubated with biotin-conjugated anti-IFN-γ detection antibody at room temperature. for 2 h, followed by incubation with streptavidin-HRP (1:1000) in PBS-0.5% FCS at r.t. for 1 h. Then, ready-to-use TMB substrate solution was added as 100 μl/well and it was developed until distinct spots emerged. Color development was stopped by washing extensively in deionized water. The plate frame was removed from the plastic tray, and the underside of the membrane was rinsed. Leave the plate to dry. Spots were imaged and quantified with a CTL ImmunoSpot Analyzer (Cellular Technology Ltd, USA).

### Luminex detection of cytokines in plasma

Cytokines in plasma were quantified with Luminex (RND, lxsahm-08) according to the instructions. Briefly, the kit was removed from the refrigerator and equilibrated in R.T. for 30 min. Prepare standards, beads, detection antibodies and PE streptavidin along with the washing solution. After resuspension of the beads, 50 μl of the diluted beads was added to each well. According to the arrangement before the experiment, 50 μl of standard substance and sample were added to each well, sealed with parafilm, and placed on a shaker at 800 rpm for 2 h. Then, the microplate was placed on the magnetic frame for 1 min and cleaned with washing solution 3 times with 100 μl per well. Fifty microliters of diluted biotin-labeled detection antibody complex was added and sealed and incubated at 800 rpm for 1 h. Place the microplate on the magnetic frame for 1 min again and wash it with 100 μl washing solution 3 times per hole. Diluted streptavidin-labeled PE was added to the corresponding wells in a volume of 50 μl per well and sealed before being placed in a shaker at 800 rpm for 0.5 h. The microplate was placed on the magnetic frame for 1 min and washed 3 times with 100 μl per hole. Finally, the beads were resuspended in 100 μl washing buffer and incubated on a shaking table for 2 min. The shaking table speed was set to 800 rpm and detected on the upper computer (Luminex X-200).

### Detection of IFN-γ and IL-2 in plasma by ELISA

Plasma IFN-γ and IL-2 were quantified using commercial ELISA kits for human IFN-γ (Biolegend, no. 430107) and IL-2 (R&D Biosystems, no. VAL110). Optical density (OD) was detected using a Gen5 microplate reader (BioTek, Vermont, USA) according to the manufacturer's instructions.

### AKT/GSK3β signal detection

COVID-19 convalescent PBMCs were inoculated in a six-well plate and grown to 60–70% confluence. Then, the cells were stimulated with anti-CD3/CD28 for 48 h. According to the manufacturer's instructions, an ELISA kit was used to quantify total Akt and phosphorylated Akt (ab126433, Abcam) and GSK3β and phosphorylated GSK3β (ab205711, Abcam). Unexposed elder PBMCs and COVID-19 convalescent PBMCs were inoculated in six-well plates and cultured to 60–70% confluence. The cells were transferred to 96-well plates coated with anti-CD3/CD28 for 48 h. Unexposed elder was treated with AKT agonist (SC79, 10 μM, 30 min), GSK3β inhibitor (TWS119, 10 μM, 24 h), and COVID-19 convalescent was treated with AKT inhibitor (GDC-0068, 10 μM, 15 min) and GSK3β agonist (sodium nitroprusside, 50 μM, 30 min, HY-0564), for a total of four groups, and finally flow cytometry was performed. SC79, TWS119, and GDC-0068 were purchased from Selleck.

### Statistical analysis

Statistical analysis of the data was performed using SPSSS 26 software. Single factor analysis of variance was used for multiple groups (≥ 3), and the post LSD method in single factor analysis of variance was used for pairwise comparison. Independent sample *t*-test were used for comparisons between the two groups. The Wilcoxon signed rank test was used to compare the data between two paired groups, and the Mann‒Whitney *U* test was used to compare the data between two unpaired groups.

### Ethics approval and consent to participate

The study was approved by the ethics committee of the First Affiliated Hospital of Nanjing Medical University (2020-SR-134). All methods were carried out in accordance with relevant guidelines and regulations. The written informed consent was obtained from all patients.

## Results

### General clinical information of COVID-19 patients

The clinical characteristics of recovered COVID-19 patients, sex-matched unexposed younger and sex/age-matched unexposed elder groups are summarized in Table 1. The average age of the convalescents was 50.84 ± 12.94 years old, with 22 (59.5%) males and 15 (40.5%) females. Among them, 2 cases (5.4%) were asymptomatic, 4 cases (10.8%) were mild, and 31 cases (83.8%) were common. The most common comorbidity was hypertension (27%), followed by type 2 diabetes (8.1%) and hyperlipidemia (5.4%). Eight patients with no previous hypertension had elevated blood pressure during follow-up. Twenty-four patients (64.9%) complained of no sequelae after discharge, 7 patients (18.9%) complained of fatigue, and 3 patients (8.1%) complained of limb numbness/restlessness. Moreover, 7 cases (18.9%) may have depression (Table 2). Collectively, recovered COVID-19 patients may develop long COVID with various comorbidities.

**Table 2:** Recruiters’ sequelae symptoms and Hamilton Depression Scale (HAMD) score.

	Unexposed younger (n = 20)	Unexposed elder (n = 20)	Convalescent COVID-19 (n = 37)
Any of the following	0	2 (10%)	13 (35.1%)
Fatigue	0	2 (10%)	7 (18.9%)
Numbness/soreness of limbs	0	0	3 (8.1%)
Hair loss	0	0	1 (2.7%)
Nacha	0	0	2 (5.4%)
Expectoration	0	0	1 (2.7%)
Chest tightness	0	0	1 (2.7%)
Brain buzzing	0	0	1 (2.7%)
HAMD score			
0–8	20 (100%)	20 (100%)	30 (81.1%)
9–19	0	0	7 (18.9%)
20–34	0	0	0
≥ 35	0	0	0

0–8 points, normal; 9–19 points, there may be depression; 20–34 points, there must be depression; 35 points or more, severe depression.

### Anti-CD3/CD28-activated T cells in convalescent COVID-19 patients

**Figure 1:**
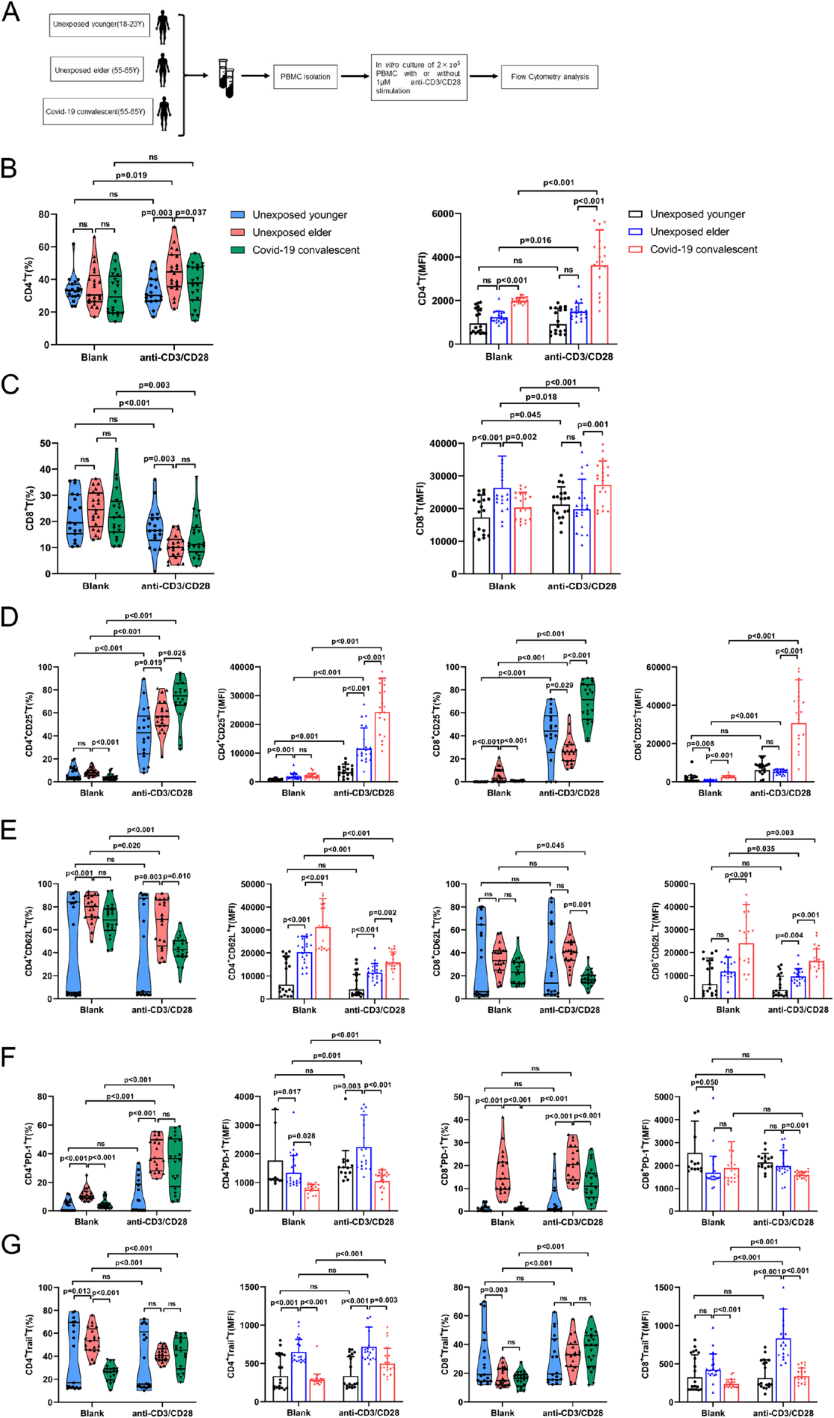
Paradoxical activation of T cells from COVID-19 convalescent patients. (**A**) PBMC processing protocol: PBMCs from subjects were extracted separately and divided into two groups: the blank group (i.e., group B) and the positive control group (group P, anti-CD3/CD28, 1:1000). Anti-CD3/CD28 stimulatory activation was performed overnight in 96-well plates. After seeding cells at a density of 2 × 10^5^ cells/well, cells were collected for detection after culturing for 48 h. (**B**,**C**) PBMCs under nonspecific activation conditions. The violin chart shows that CD4^+^ T cells are more easily activated in the elderly group, while CD8^+^ T cells are more easily activated. Compared with unexposed elderly individuals, COVID-19 convalescent CD4^+^ T-cell activation was reduced, and CD8^+^ T cells were not significantly different. In addition, in the Blank group, there was no significant difference in the percentage of CD4^+^ T cells and CD8^+^ T cells between the unexposed elder group and the COVID-19 recovered patients. (**D**–**G**) Under nonspecific stimulation conditions, unexposed younger, unexposed elder and COVID-19 convalescent T-cell activation: compared with unexposed younger, unexposed elder PD-1 and CD62 L have higher expression in CD4^+^ T and CD8^+^ T (% and MFI), while the frequency of CD4^+^CD25^+^ T increases, the frequency of CD8^+^CD25^+^ T decreases, and MFI values were significantly higher in both CD4^+^Trail^+^ T and CD8^+^Trail^+^ T cells. Compared with unexposed elderly individuals, COVID-19 convalescent CD25^+^CD4^+^ T cells and CD25^+^CD8^+^ T cells increased (% and MFI), while the frequencies of CD4^+^CD62 L^+^ T cells and CD8^+^CD62 L^+^ T cells decreased. The frequency and MFI of CD8^+^PD-1^+^ T cells also decreased significantly. At the same time, TRAIL expression appeared significantly lower (MFI) in COVID-19 convalescent CD4 T and CD8 T cells. No significant differences were observed in the remaining indexes. Color violin plots are statistical analysis plots for cell proportions, and column plots are MFI statistical analysis plots. Blue for unexposed elder, COVID-19 convalescent in red (n = 20).

To explore the immune profiles of T lymphocytes, unexposed younger individuals, age-matched unexposed elderly individuals and convalescent COVID-19 PBMCs were given anti-CD3/CD28 activation stimulation (Fig. 1A). Comparing the age-matched Unexposed elder and COVID-19 convalescent, we found that the frequency of CD4^+^ T lymphocytes was lower in the COVID-19 convalescent than in the unexposed elder, and the frequency of CD8^+^ T cells did not change significantly (Fig. 1B,C). Moreover, CD4^+^CD25^+^ T and CD8^+^CD25^+^ T cells (% and MFI) were increased in COVID-19 convalescent patients (Fig. 1D), while the frequency of CD4^+^CD62L^+^ T and CD8^+^CD62L^+^ T cells was decreased (Fig. 1E), suggesting that T cells in COVID-19 convalescent patients may be prone to activation. In contrast, CD8^+^PD-1^+^ T cells (Fig. 1F) were significantly decreased in convalescent COVID-19 patients. Although the immune profiles of T cells from convalescent COVID-19 patients were different from those of T cells from age-matched unexposed elderly patients, it was surprising that the expression of surface markers, including CD4, CD8, CD25, PD-1 and TRAIL, was similar between convalescent COVID-19 patients and unexposed younger patients (Fig. 1G). Collectively, we demonstrated that upon activation with anti-CD3/CD28, T-cell immune profiles in COVID-19 patients who recovered for 6 months may still be in a potentially hypersensitive state.

### SARS-CoV-2 S/N protein exhausted T cells in convalescent COVID-19 patients

**Figure 2:**
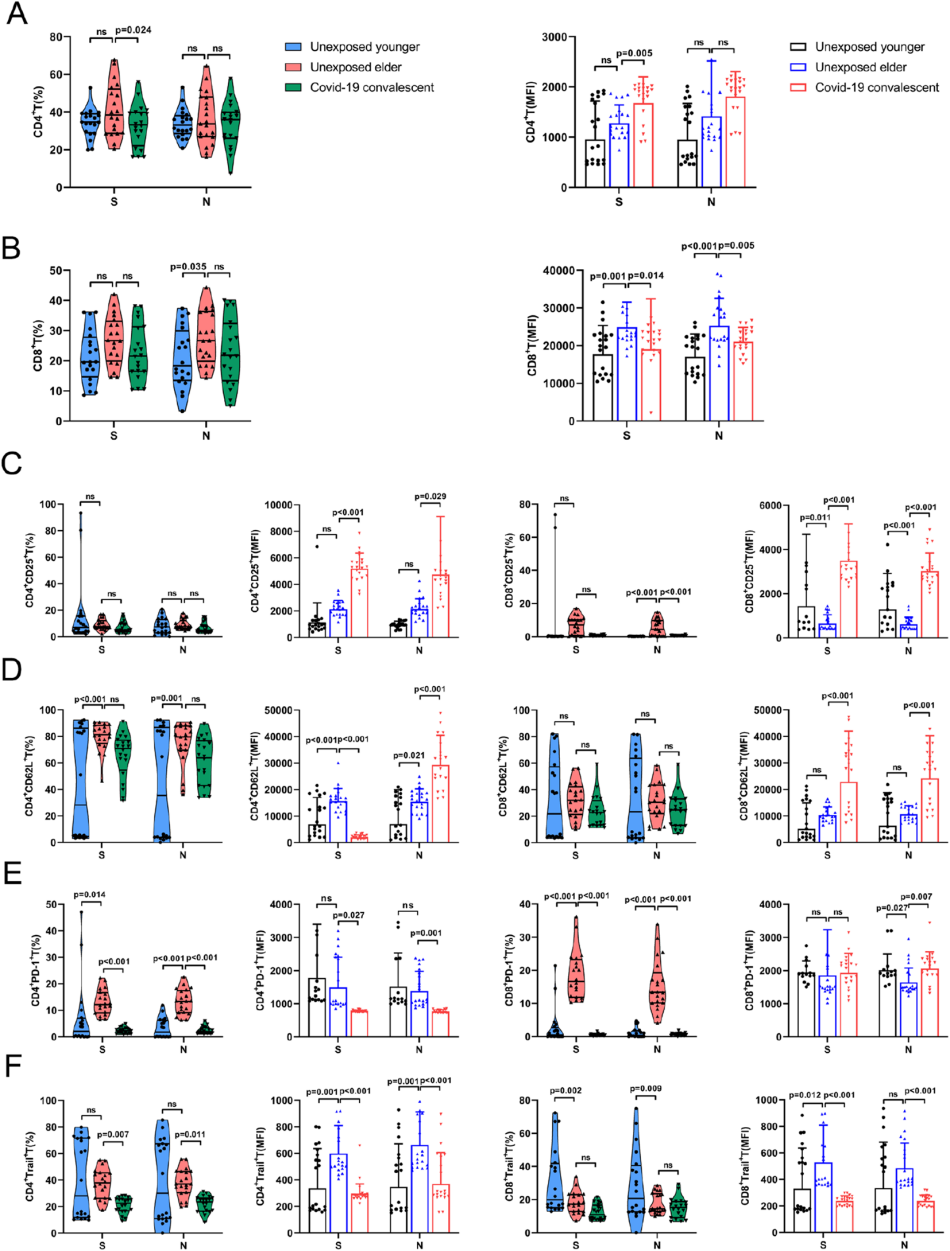
SARS-CoV-2 protein-activated T cells from COVID-19 convalescent patients. (**A**,**B**) Under S protein stimulation, compared with unexposed elderly individuals, unexposed younger CD4^+^ T cells and CD8^+^ T cells showed no significant changes, COVID-19 convalescent CD4 T-cell expression was significantly reduced, and CD8^+^ T-cell expression was not significantly different. Under N protein stimulation, compared with Unexposed elder, Unexposed younger CD8^+^T expression decreased, and CD4^+^T expression had no significant difference, while the COVID-19 convalescent CD4^+^T and CD8^+^T expression had no statistically significant difference. (**C**–**F**) Under S protein stimulation, in the unexposed elder compared with the unexposed younger, the CD4^+^CD62 L^+^, CD4^+^PD-1^+^T and CD8^+^PD-1^+^T frequencies increased, while the CD8^+^Trail^+^T frequency decreased; in the COVID-19 convalescent compared to the unexposed elder, the frequencies of PD-1^+^, Trail^+^CD4^+^T and CD25^+^, PD-1^+^CD8^+^T were all reduced. Under stimulation with the N protein, the expression of CD62 L^+^ and PD-1^+^CD4^+^ T cells and CD25^+^ and PD-1^+^CD8^+^ T cells in unexposed elderly individuals was increased compared with that in younger individuals. The expression of CD8^+^Trail^+^ T cells decreased, while the expression frequency of PD-1^+^, Trail^+^CD4^+^ T cells and CD25^+^ PD-1^+^CD8^+^ T cells in COVID-19 convalescent individuals was reduced compared with that in unexposed elderly individuals. There was no significant difference in the rest (n = 20).

Due to the mutation of SARS-CoV-2, reinfection is a new challenge. To examine the T-cell immune status during COVID-19 convalescent reinfection with SARS-CoV-2, we stimulated PBMCs from each of the three groups of samples with the SARS-CoV-2 S protein and N protein. Under N protein stimulation, convalescent COVID-19 patients had no significant changes in the total number of CD4^+^ T and CD8^+^ T cells compared with the unexposed elderly patients; meanwhile, S protein significantly decreased the percentage of CD4^+^ T cells in the convalescent COVID-19 patients (Fig. 2A,B). The mean fluorescence intensity (MFI) of CD25 in CD4^+^ T and CD8^+^ T cells upon stimulation with either S or N protein was significantly increased in convalescent COVID-19 patients (Fig. 2C). The CD62L expression level in CD4^+^ T cells stimulated with the S protein almost vanished. In contrast, CD62L was increased in CD4^+^ T cells upon N protein stimulation or in CD8^+^ T cells upon either S or N protein stimulation (Fig. 2D). PD-1 and TRAIL, two molecules associated with immune suppression, were increased in CD4^+^ T and CD8^+^ T cells upon stimulation with either the S or N protein (Fig. 2E,F). Similar to nonspecific anti-CD3/CD28 stimulation, SARS-CoV-2 S and N proteins may also lead to a state of immune exhaustion in T lymphocytes, with increased CD25 and CD62L and decreased PD-1 and TRAIL in convalescent COVID-19 patients. A previous study showed that SARS-CoV-2 may exhaust T cells in acute infection^32^. We demonstrated that even in convalescent COVID-19 patients, T cells may still be in a preexhausted state.

### Elevated plasma IFN-γ in convalescent COVID-19 patients

**Figure 3:**
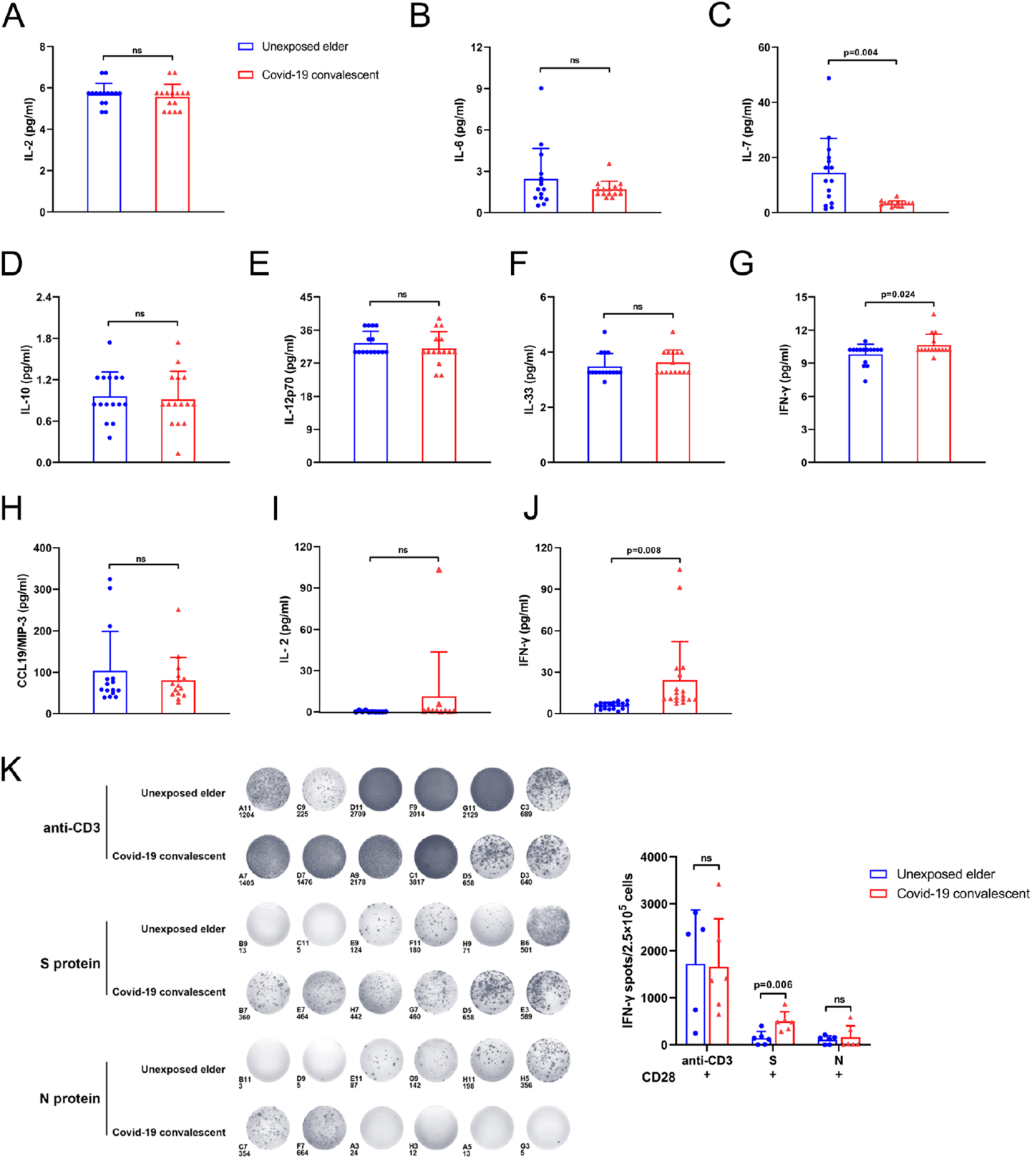
IFN-γ was increased in the plasma from COVID-19 convalescent patients. Luminex and ELISA detection of unexposed elderly individuals and COVID-19 convalescent plasma cytokines. (**A**–**H**) is Luminex detection of cytokines related to PD-1 in the elder group and COVID-19 convalescent plasma, including IL-2, IL-6, IL-7, IL-10, IL-12p70, IL-33, IFN-γ, CCL19/MIP-3. The bar chart shows that, compared with unexposed elderly individuals, COVID-19 convalescent inflammatory factor IFN-γ secretion increased, while IL-7 secretion decreased, and there was a significant difference. There was no significant difference in the rest (n = 15). (**I**,**J**) ELISA was used to compare the expression of IL-2 and IFN-γ in the plasma of the two groups [(**I**) n = 10; (**J**) n = 18]. Figures (**G**) and (**J**) show two different methods for detecting the amount of IFN-γ in plasma, and the results of the two methods are consistent. (**K**) Unexposed elderly and COVID-19 convalescent PBMCs under anti-CD3/CD28, S protein and N protein stimulation. ELISPOT detected the secretion of IFN-γ. Cells were seeded at a density of 2 × 10^5^ cells/well, and the activation time was 48 h. Statistical analysis of the number of spots in different ELISPOT wells shows that under S protein stimulation conditions, COVID-19 convalescent T cells can secrete more IFN-γ (n = 6).

PD-1 is a master immune checkpoint molecule that may promote immune exhaustion^33^. To explore the mechanisms of decreased expression of PD-1 in CD4^+^ and CD8^+^ T cells from convalescent COVID-19 patients, we measured plasma cytokines associated with PD-1 expression, including IL-2, IL-6, IL-7, IL-10, IL-12p70, IL-33, IFN-γ and CCL19/MIP-3, by Luminex. Compared with age-matched unexposed elderly individuals, convalescent COVID-19 patients presented with more IFN-γ and less IL-7, while IL-2, IL-6, IL-10, IL-12p70, IL-33 and CCL19/MIP-3 were unchanged (Fig. 3A–H). The levels of IL-2 and IFN-γ were further validated by ELISA (Fig. 3I,J), confirming that IFN-γ in COVID-19 convalescent plasma was significantly increased.

T cells are major producers of IFN-γ. In the nonspecific activation with anti-CD3 and anti-CD28, IFN-γ was robustly secreted from T cells from age-matched unexposed controls or convalescent COVID-19 patients. Specifically, stimulated with SASRS-CoV-2 S protein but not N protein, T cells only from convalescent COVID-19 patients produced IFN-γ in the ELISPOT assay (Fig. 3K). Considering that these convalescent COVID-19 patients were negative for SARS-CoV-2, we hypothesized that SARS-CoV-2 antibodies mimicking the virus may be persistent and keep evoking the production of IFN-γ^34^. In summary, plasma IFN-γ levels in convalescent COVID-19 patients after 6 months were still higher.

### IFN-γ suppressed PD-1 in T cells from convalescent COVID-19 patients

**Figure 4:**
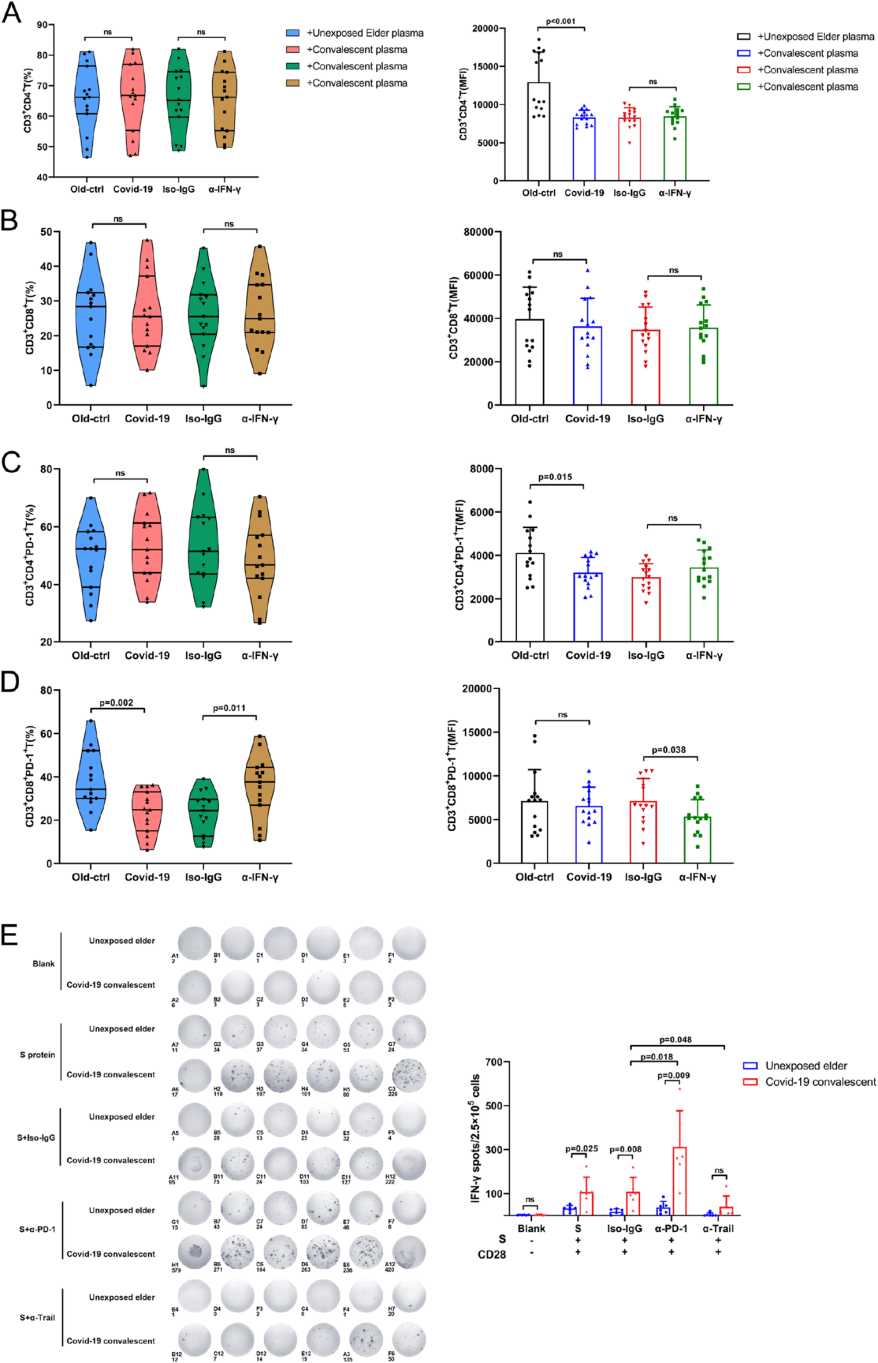
The reciprocal regulation between IFN-γ and PD-1 in T lymphocytes. (**A**–**D**) Unexposed younger PBMCs were cocultured with unexposed elder plasma and COVID-19 convalescent plasma to detect PD-1 expression on T cells. In addition, an IFN-γ neutralizing antibody was also used to verify the effects of IFN-γ on CD8^+^PD-1^+^ T-cell expression. The results showed that the expression frequency of PD-1^+^CD8^+^ T cells in the COVID-19 convalescent plasma group was lower than that in the unexposed elder plasma group. At the same time, the expression frequency of CD8^+^PD-1^+^ T cells in the IFN-γ neutralizing antibody group was significantly higher than that in the isotype control group (iso-IgG), while the CD4^+^ T, CD8^+^ T and CD4^+^PD-1^+^ T-cell expression frequencies were not significantly different. The difference in the expression of some indexes of MFI is more likely to reflect the state of individual cells at that time (n = 15). (**E**) Unexposed elder or COVID-19 convalescent PBMCs were cocultured with S protein, iso-IgG, PD-1, and trail neutralizing antibodies. Under S protein stimulation, the three groups of cells secreted IFN-γ. Column graphs show that compared with the isotype control group, the COVID-19 convalescent with α-PD-1 group had more IFN-γ secretion than the unexposed elder group. Furthermore, the COVID-19 convalescent with added Trail neutralizing antibody also promoted a decrease in IFN-γ secretion (n = 6).

To explore the potential roles of IFN-γ in the expression of PD-1, PBMCs from healthy controls were treated with unexposed elder plasma or convalescent COVID-19 plasma in combination with IFN-γ neutralization antibody or isotype control antibody. As shown in Figure 4A,B, the expression of CD4 and CD8 was similar, with the exception that convalescent COVID-19 plasma significantly decreased the MFI of CD4. Regarding PD-1 expression (Fig. 4C,D), convalescent COVID-19 plasma decreased PD-1 in CD4^+^ T cells (MFI) and in CD8^+^ T cells (%); moreover, anti-IFN-γ treatment rescued the expression of PD-1 in CD8^+^ T cells (%), suggesting that IFN-γ in convalescent COVID-19 plasma may inhibit the expression of PD-1 on CD8^+^ T cells.

IFN-γ reciprocally interacted with PD-1^29,30^. To explore whether PD-1 expression on T cells contributed to the higher IFN-γ in convalescent COVID-19 plasma, we treated PBMCs from unexposed elder or convalescent COVID-19 patients with S protein in combination with PD-1 neutralization antibody or isotype control antibody (Fig. 4E). S protein significantly boosted IFN-γ production in convalescent COVID-19 PBMCs. Once blocked with a PD-1 neutralization antibody, IFN-γ production in convalescent COVID-19 PBMCs was further increased. In contrast, the TRAIL neutralization antibody did not affect the production of IFN-γ. Collectively, elevated plasma IFN-γ in convalescent COVID-19 patients may decrease the expression of PD-1 in T cells, which reciprocally boosted the production of IFN-γ.

### Bioinformatics analysis revealed that AKT/GSK3β may regulate PD-1 expression in T cells from COVID-19 convalescent patients

**Figure 5:**
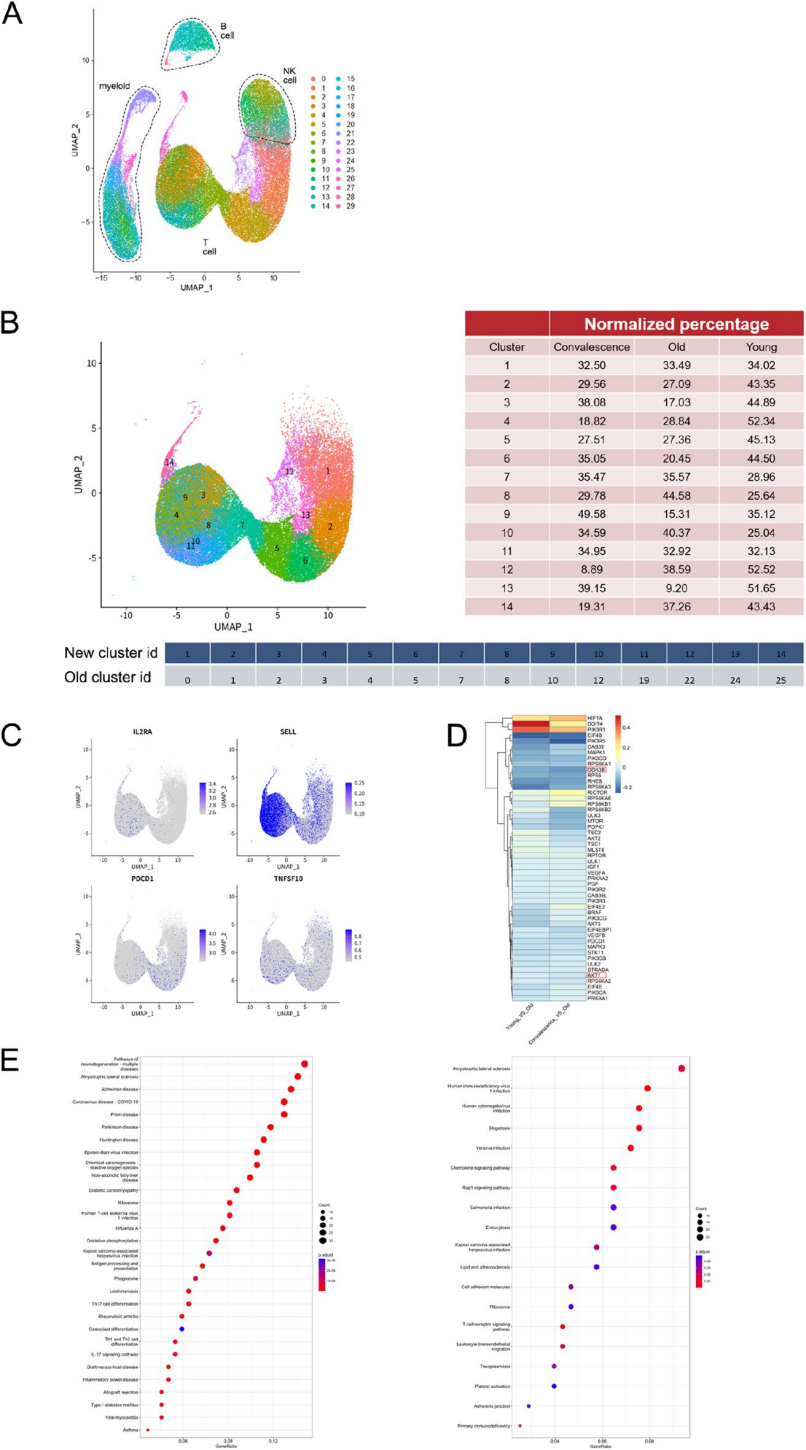
Bioinformatics revealed the T-cell subpopulation and possible mechanisms. (**A**) Selected samples from the article that met this experiment for bioinformatics analysis, including 10 cases of unexposed younger, 3 cases of unexposed elder, and 4 cases of COVID-19 rehabilitation samples. The UMAP dimension reduction plot is displayed with a table. (**B**) Identifying cell clusters based on enriched genes and quantifying their relative abundance. (**C**) Gene expression plots showing the distribution of CD25 (IL2RA), CD62 L (SELL), PD-1 (PDCD1) and Trail (TNFSF10). Expression levels for each cell are shown as Pearson residuals and displayed using a color scale overlaid onto the UMAP plot. (**D**) Heatmap displaying hierarchical cluster analysis of PD-1 and mTOR pathway mRNA between unexposed younger vs unexposed elder and unexposed elder vs COVID-19 convalescent. The color scale indicates intensity increases from blue to red, which indicates down- and upregulation, respectively. (**E**) KEGG signaling pathway database analysis of the corresponding signaling pathways of differentially expressed genes between unexposed younger vs unexposed elder and unexposed elder vs COVID-19 convalescent.

To further study the possible related signaling pathways by which IFN-γ regulates PD-1 expression on CD8^+^ T cells, we used the single-cell sequencing results of the COVID-19 samples studied by Ren et al.^35^. We collected samples that were consistent with this experiment, including 10 cases of unexposed younger individuals, 3 cases of unexposed elderly individuals and 4 cases of COVID-19 convalescent samples. First, cluster analysis showed that PBMCs mainly included four types of cell enrichment: B cells, T cells, NK cells and myeloid cells (Fig. 5A). Based on enriched genes and quantifying their relative deficiency, 14 T-cell subsets were identified (Fig. 5B). Gene expression plots showing the distribution of CD25 (IL2RA), CD62 L (SELL), PD-1 (PDCD1) and Trail (TNFSF10) in T cell clusters (Fig. 5C). According to the differentially expressed genes of unexposed elderly individuals and COVID-19 convalescent individuals, the signaling pathways related to differentially expressed genes were analyzed by immunologic signature enrichment analysis in the KEGG database (Fig. 5D,E). The downregulated signaling pathways included amyotrophic lateral sclerosis, human immunodeficiency virus 1 infection, human cytomegalovirus infection, and shigellosis (count ≥ 20, P < 0.01). The upregulated signaling pathways included neurogeneration multiple diseases, amyotrophic lateral sclerosis, Alzheimer's disease, and coronavirus disease-COVID-19 (count > 20, P < 1e−04). In particular, genes in the mTOR pathway^31,36,37^ may be correlated with PD-1 expression; specifically, GSK3β had a significant positive correlation with PD-1, while AKT had a certain negative correlation.

### IFN-γ reduced PD-1 on CD8^+^ T cells via the AKT/GSK3β signaling pathway

**Figure 6:**
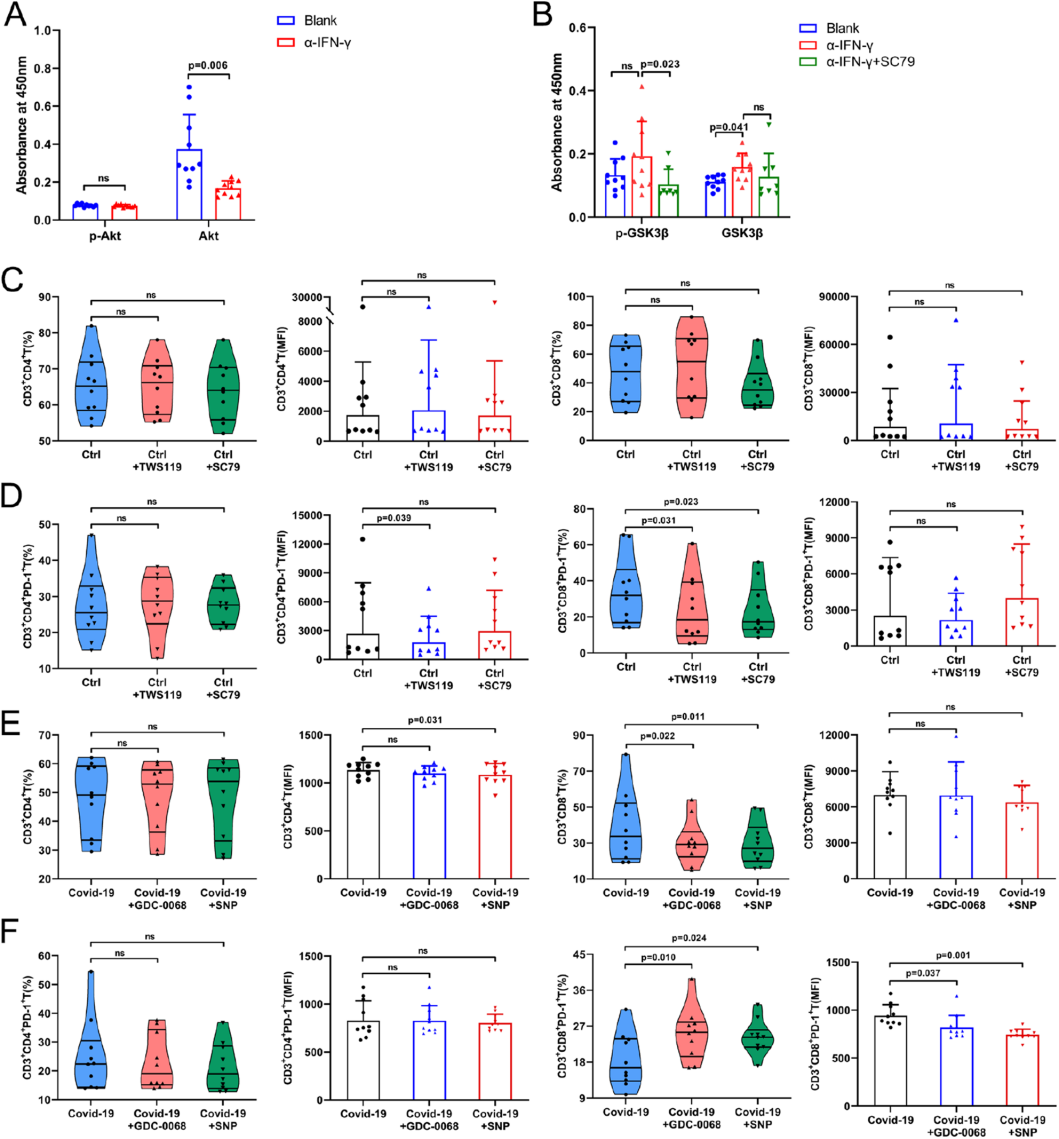
IFN-γ downregulated PD-1 through the AKT/GSK3β signaling pathway. (**A**,**B**) COVID-19 convalescent PBMCs were cultivated, and ELISA was used to detect the expression of p-AKT, p-GSK3β and total AKT and GSK3β proteins in different groups of cells. SC79 is an AKT agonist. The bar graph results show that compared with the control group, the expression of AKT in the α-IFN-γ group was lower, and GSK3β was increased, but there was no significant difference in the expression of p-AKT and p-GSK3β. This finding indicates that IFN-γ positively regulates AKT and negatively regulates GSK3β. After adding SC79, we found that compared with the α-IFN-γ group, the p-GSK3β of the SC79 group was significantly reduced, but there was no significant difference in total GSK3β. To some extent, these findings show that AKT has a negative regulatory effect on GSK3β. (**C**,**D**) Flow cytometric detection of T-cell activation after unexposed elder PBMCs were cocultured with an AKT agonist (SC79) and a GSK3β inhibitor (TWS119). The violin chart shows that under the two different culture conditions, the expression of CD8^+^PD-1^+^ T cells was significantly different from that of the blank group. (**E**,**F**) Flow cytometric detection of T-cell activation after COVID-19 convalescent PBMCs were cocultured with an AKT inhibitor (GDC-0068) and a GSK3β agonist (SNP). The violin chart shows statistically significant differences in CD8^+^PD-1^+^ T-cell expression compared with the blank in both culture conditions. No significant differences were observed in the remaining indexes (n = 10).

According to the abovementioned bioinformation analysis, we speculated that IFN-γ may regulate the expression of PD-1 on CD8^+^ T cells by regulating the AKT/GSK3β signaling pathway. Compared with the isotype control group, the AKT level was reduced in PBMCs upon α-IFN-γ treatment (Fig. 6A). In contrast, the expression of GSK3β increased, while p-Akt and p-GSK3β expression was not significantly different. When an AKT agonist (SC79) was added, we found that compared with the α-IFN-γ group, the p-GSK3β of the α-IFN-γ + SC79 group was significantly reduced, but there was no significant difference in total GSK3β, confirming the negative regulation of GSK3β by AKT (Fig. 6B). Furthermore, we cocultured unexposed aged PBMCs with an AKT agonist (SC79) and a GSK3β inhibitor (TWS119) while administering T-cell activation. The results showed that the expression of CD8^+^PD-1^+^ T cells was significantly different from that in the blank group under these two different culture conditions (Fig. 6C,D). In addition, PBMCs from the COVID-19 recovery group were cocultured with an AKT inhibitor (GDC-0068) and a GSK3β agonist (SNP) and stimulated with T-cell activation. The violin diagram showed that the expression of CD8^+^PD-1^+^ T cells under the above two different stimulation conditions was significantly different from that of the blank group (Fig. 6E,F). In summary, IFN-γ reduced PD-1 on CD8^+^ T cells at least partially by the AKT/GSK3β signaling pathway.

## Discussion

Cellular immune responses play critical roles in viral clearance and disease severity^3,38,39^. CD8^+^ T cells are important in eliminating large numbers of virus-infected cells during severe infection^40^. The timing of T-cell response protection may greatly affect the prevalence of the pandemic^41^. The number of functionally impaired T cells is significantly reduced in COVID-19 patients, especially in severe cases^42–44^. Furthermore, extensive phenotypic alterations and underlying dysfunction remain prominent in T cells of individuals clinically recovering from COVID-19^45^. SARS-CoV-2-specific T cells drop dramatically within one month of clinical recovery^46^. In the present study, we recruited 43 Wuhan COVID-19 patients who recovered 6 months. Phenotypic analysis studies showed that there was no significant difference in the percentage of CD4^+^ T cells and CD8^+^ T cells between the unexposed elder group and the COVID-19 convalescent patients. However, either under nonspecific stimulation conditions or under specific S protein and N protein stimulation conditions, both CD4^+^ T cells and CD8^+^ T cells in the unexposed elder group showed increased activation indicators to a certain extent. In specific, PD-1 expression in CD4^+^ T cells or CD8^+^ T cells was reduced in the COVID-19 convalescent patients (Figs. 1F, 2E), which was in line with the previous exploring T cells from patients 6 months post COVID-19 convalescence^47^.

Compared with the unexposed-elder group, IFN-γ in the plasma of COVID-19 convalescent patients was higher (Fig. 3J), which may inhibit the expression of PD-1 on CD8^+^ T-cell membranes of COVID-19 convalescent patients. Meanwhile, PD-1 inhibited the secretion of IFN-γ by T cells in COVID-19 convalescent patients (Fig. 4E). This has also been confirmed in acute infection of bovine viral diarrhea virus (BVDV)^48^. Bioinformatics analysis showed that there was a significant positive correlation between PD-1 and GSK3β in the mTOR pathway, and there was a certain negative correlation with AKT. Signaling pathway studies confirmed that IFN-γ could reduce the expression of PD-1 on CD8^+^ T cells by regulating the AKT/GSK3β signaling pathway (Fig. 6). AKT/GSK3β signaling pathway regulates PD-L1 in cancer cells^49^ and PD-1 in chimeric antigen receptor T (CAR-T) lymphocytes^50^. AKT inhibitor rescued the expression of PD-1 in CD8^+^ T cells (Fig. 6F). Recently, the first AKT inhibitor capivasertib has been approved to treat breast cancer^51^. It is interesting to ask whether capivasertib and other drugs targeting AKT or GSK3β could be used to alleviate long COVID.

There are some limitations in the current study. This study only focused on a population that had been infected with the Wuhan SARS-CoV-2 rehabilitation and recovered patients for 6 months. There is a lack of comparison of CD8^+^PD-1^+^ T expression data in PBMCs of the population during the acute phase of virus infection and other rehabilitation periods. Studies have confirmed that recovery from COVID-19 has distinct phenotypic characteristics that correlate with the severity of COVID-19^52^. Furthermore, research on immunotherapy for the plasma of the current recovered COVID-19 patients^32,53^ should combine antibody therapy in the humoral immune response to the study of T-cell phenotype mechanisms. In addition, assessment of immune markers can help predict prognosis and disease severity in COVID-19 patients. Of note, expression of PD-1 in our study was measured by MFI and percentage, which was not always synchronous at any time. MFI measures the amounts of PD-1 molecules in the T lymphocytes. Percentage indicates the proportion of PD-1 positive cells in the T lymphocytes. The plausible clue for the PD-1 inconsistence in MFI and percentage is unknown. More markers are needed to provide clues for the clinical diagnosis, treatment, and prognostic evaluation of COVID-19. Finally, the cohort size of the present study is small. Due to the strict control in Wuhan COVID-19, we could not recruit a large set of patients recovered from the Wuhan strain. Of note, 15 recovered COVID-19 patients, smaller than our cohort, may still provide valuable information regarding to the dysfunctional B lymphocytes^54^. As similar, in the present study we explored T lymphocytes over 30 recovered COVID-19 patients.

In sum, recovered COVID-19 patients from Wuhan SARS-CoV-2 may develop long COVID. Increased IFN-γ in the plasma of recovered COVID-19 patients contributed to PD-1 downregulation on CD8^+^ T cells, which was regulated by the AKT/GSK3β signaling pathway. Aberrant IFN-γ in the plasma and PD-1 in CD8^+^ T cells may indicate the dysfunctional immune status post COVID-19 infection.

## Data Availability

The data that support the findings of this study are available from the corresponding author upon reasonable request.
